# Eagle’s Syndrome Managed Successfully by Pulsed Radiofrequency Treatment

**DOI:** 10.7759/cureus.10574

**Published:** 2020-09-21

**Authors:** Bhanu P Swain, Sri Vidhya, Sharad Kumar

**Affiliations:** 1 Anesthesiology, Tata Main Hospital, Jamshedpur, IND

**Keywords:** eagle’s syndrome, glossopharyngeal neuralgia, pulsed radiofrequency treatment, styloid process, craniofacial pain, oropharyngeal pain, otalgia

## Abstract

Eagle’s syndrome is a rare cause of craniofacial pain caused by impingement of adjacent neurovascular elements by an elongated styloid process or by a calcified stylohyoid ligament. There is a wide spectrum of clinical presentations, which encompasses craniofacial pain, oropharyngeal pain, otalgia, headache, and vertigo. Typically, the glossopharyngeal nerve gets entrapped, giving rise to characteristic orofacial pain. The diagnosis of Eagle’s syndrome is confirmed radiologically, and the management includes pharmacotherapy and surgical removal of the styloid process. Moreover, minimally invasive interventions in the form of glossopharyngeal nerve block and radiofrequency treatment can also be effective in providing pain relief. We report a case of an elderly male who presented with features of glossopharyngeal neuralgia secondary to an elongated styloid process and was managed successfully with pulsed radiofrequency treatment of the glossopharyngeal nerve.

## Introduction

Watt Weems Eagle, an American otolaryngologist, first described Eagle’s syndrome as a rare cause of craniofacial pain due to abnormal elongation of the styloid process [1]. The clinical manifestation is due to the compression of the adjoining neurovascular structures by the anomalous bony growth. Most commonly, the glossopharyngeal nerve is involved, leading to pain in the throat and neck radiating to the ear [2]. Infrequently, the styloid process may impinge on carotid vessels and other neural elements nearby, leading to a host of symptoms such as syncope, bradycardia, facial pain, and hoarseness of voice [3].

In the general population, the length of the styloid process is usually less than 2.5 cm. It is interpreted as elongated when the length is more than 3-4 cm (as seen in imaging studies), and it includes the combined length of the styloid process and the ossified stylohyoid ligament [4]. There are several hypotheses regarding the pathogenesis of Eagle’s syndrome. The classical description is the overgrowth of the styloid process or ossification of the stylohyoid ligament due to trauma during tonsillectomy surgery, as originally described by W. W. Eagle. Developmental abnormalities and insertion tendinosis of stylohyoid ligament due to degenerative changes associated with normal aging are the few other hypotheses [5].

Eagle’s syndrome is often a diagnosis of exclusion due to its heterogenous nonspecific presentation, and the diagnosis is only established by radiological studies. Treatment of Eagle’s syndrome usually begins with pharmacological agents such as anticonvulsants, antidepressants, non-steroidal anti-inflammatory drugs (NSAIDs), acetaminophen, and weak opioids such as tramadol. Surgical intervention in the form of styloidectomy is the definitive management when medical management fails [4]. Moreover, minimally invasive interventional therapy in the form of glossopharyngeal nerve block followed by radiofrequency treatment is an attractive alternative. There is only one previous case report illustrating the use of pulsed radiofrequency (PRF) treatment of the glossopharyngeal nerve in a case of Eagle’s syndrome [6].

We report a case of a patient who presented with unilateral otalgia and throat pain mimicking glossopharyngeal neuralgia, which on radiographical examination confirmed to be secondary to the elongated styloid process (Eagle’s syndrome). The patient underwent a diagnostic glossopharyngeal nerve block followed by PRF treatment, which provided excellent long-term pain relief.

## Case presentation

A 66-year-old male patient was referred to the pain clinic with a history of intense pain in the ear, angle of the mandible, and right side of the neck for four months. On further questioning, he also complained of foreign body sensation and pain in the throat on swallowing. The pain was insidious in onset and was not associated with fever, ear discharge, or upper respiratory tract infection. It was continuous, sharp, shooting, and burning in nature, with intermittent exacerbation (10-12 times a day). Chewing, swallowing, and neck movement often aggravated the pain intensity. The intensity of pain on the numerical rating scale was 8-9/10, and the pain disability score was 40 out of a maximum score of 70. There was no history of any other associated comorbidity.

The patient was initially under the treatment of otolaryngologists because of otalgia. On examination, the otolaryngologist found no sign of local infection or any visible lump in the neck, and a preliminary diagnosis of wax impaction was made. However, there was no pain relief despite wax removal, and the patient was referred to the pain clinic.

**Figure 1:**
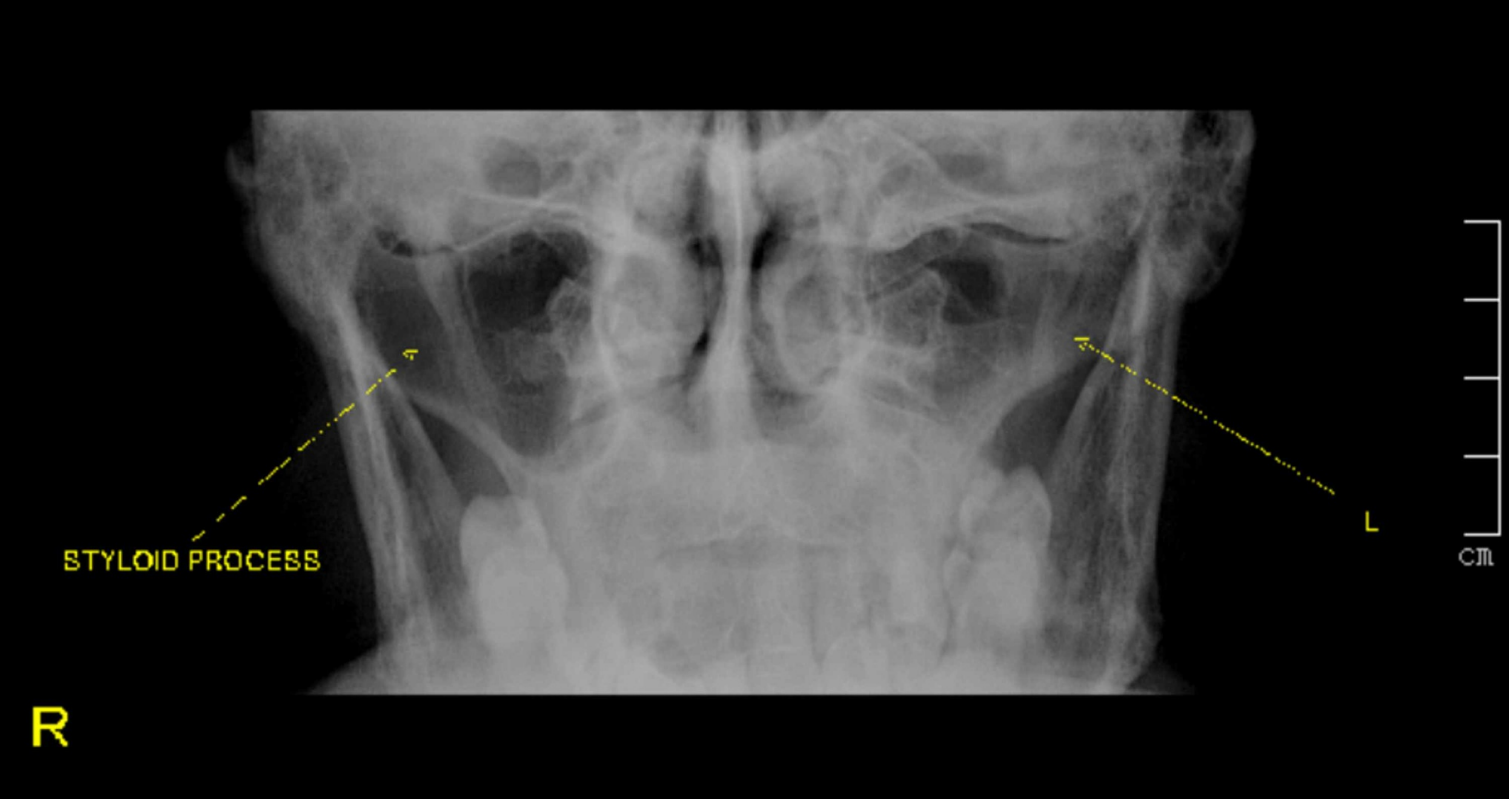
X-ray of the skull (Towne's view) showing an elongated styloid process on the right side and a normal styloid process on the left side

**Figure 2:**
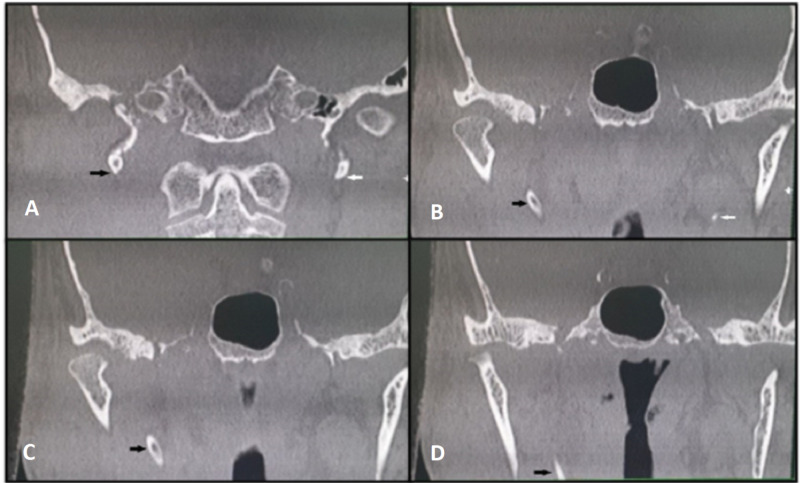
Serial coronal sections of CT scan of the head and neck Images (A-D) showing an elongated styloid process on the right side depicted by the black arrow. Note the normal styloid process on the left side (A and B) depicted by the white arrow.

In the pain clinic, a detailed examination was performed, and all the investigations were reviewed. Based on clinical presentation, a provisional diagnosis of glossopharyngeal neuralgia was made. The patient was started on carbamazepine 200 mg daily in divided dose along with paracetamol and tramadol as analgesics. There was some symptomatic relief with medication; however, he experienced excessive drowsiness, dizziness, and nausea. Other anticonvulsants and antidepressants were tried but to no avail as none of them were tolerated by the patient. Meanwhile, an X-ray of the skull was performed, which revealed an elongated styloid process of approximately 6 cm on the right side and 2.4 cm on the left side (Figure 1). The diagnosis of Eagle’s syndrome was later confirmed with computed tomography (CT) scan of the head and neck (Figure 2). Magnetic resonance imaging (MRI) scan of the brain was also performed to rule out any vascular compression or intracranial mass, which came out to be normal.

**Figure 3:**
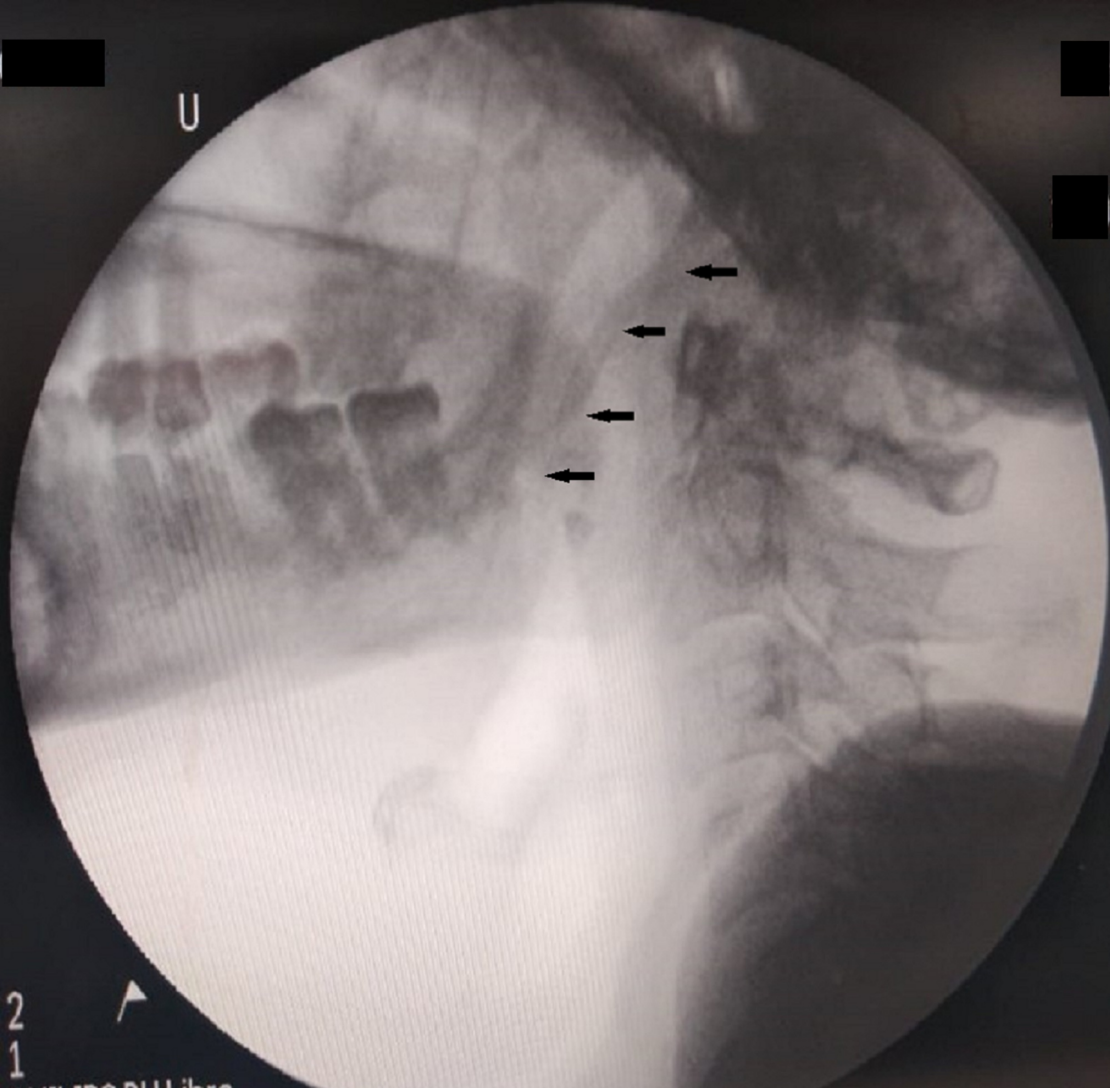
Fluoroscopic image (lateral view) showing the elongated styloid process depicted by black arrows

As medical management could not be continued due to side effects, and the patient didn’t want surgical intervention, a diagnostic glossopharyngeal nerve block was performed. The block was performed by the extra-oral approach under fluoroscopy guidance. A 2.5-inch hypodermic needle was introduced at the midpoint of the imaginary line connecting the tip of the mastoid process and the angle of the mandible to hit the styloid process (Figure 3) under direct fluoroscope vision. After contacting the styloid process, the needle was passed just behind it to elicit concordant paresthesia and then 1.5-mL 2% xylocaine with 2 mg of dexamethasone was injected. Post-procedure visual analog scale (VAS) score decreased to 2/10 from pre-procedure VAS score of 8/10. The patient reported excellent pain relief for 48 hours, and the decision was taken for PRF treatment of the glossopharyngeal nerve to provide long-term pain relief.

**Figure 4:**
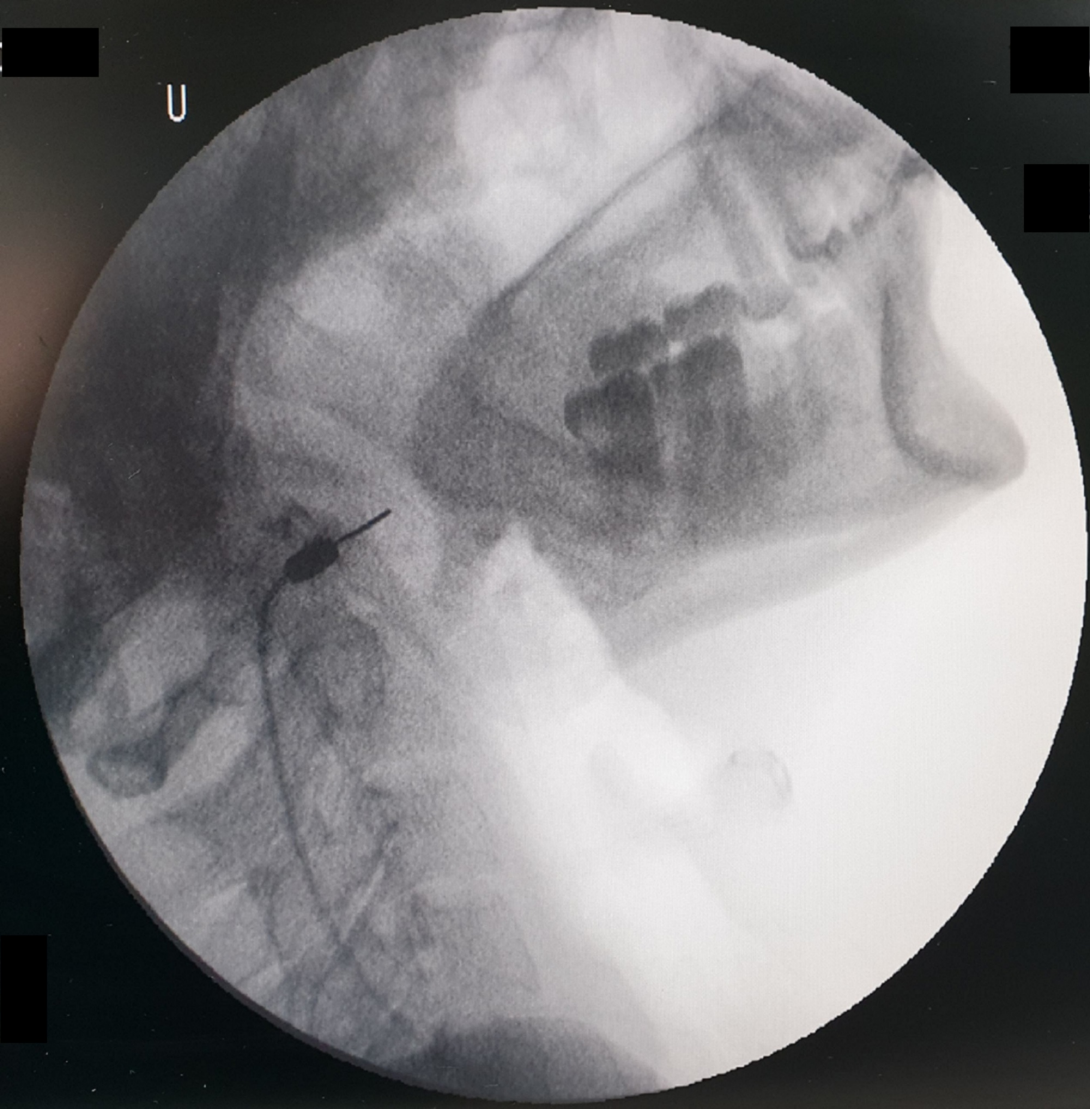
Fluoroscopic image showing the radiofrequency needle in the lateral view

**Figure 5:**
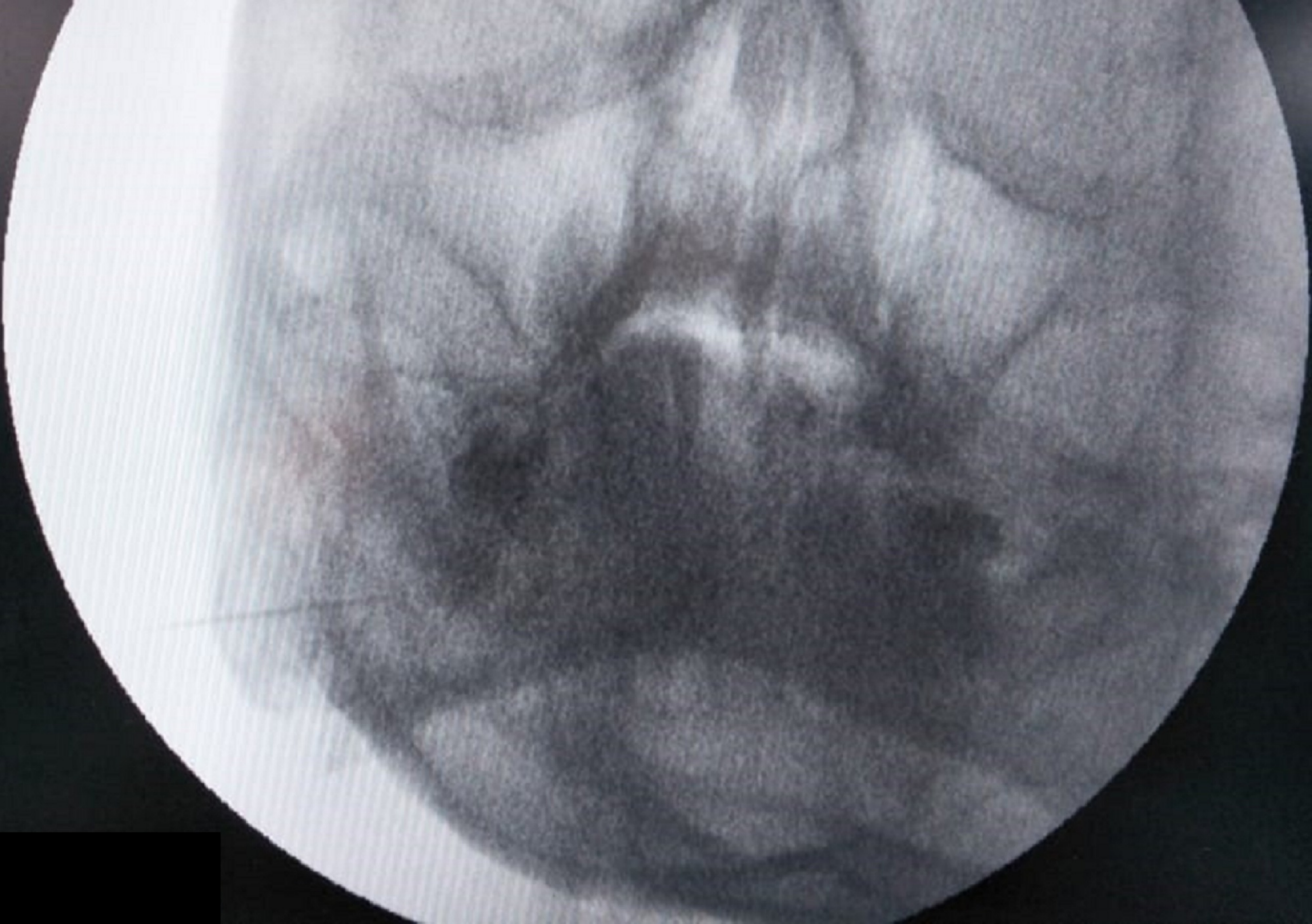
Fluoroscopic image showing the radiofrequency needle in the anterior-posterior view

**Figure 6:**
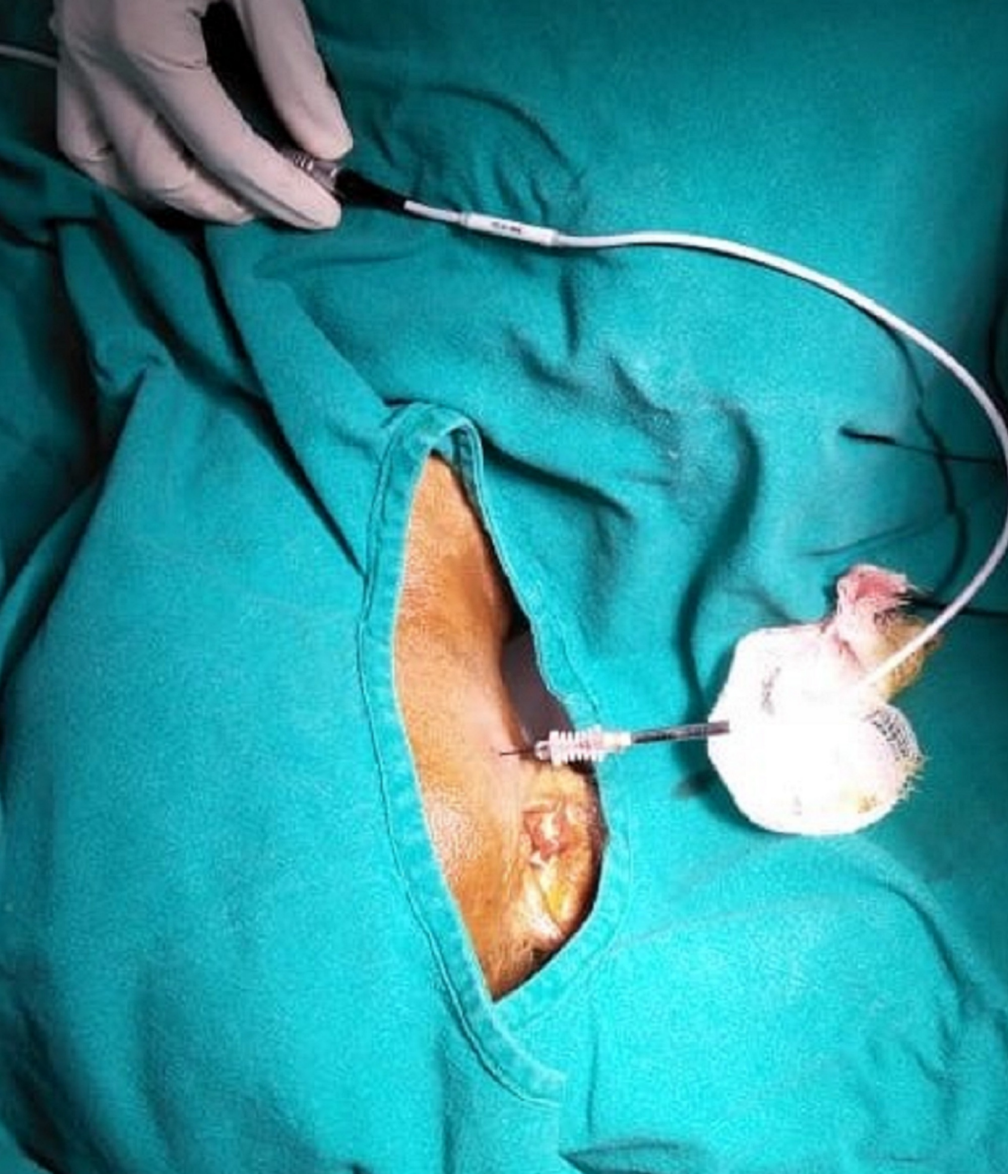
Image showing the patient position and the needle entry point during the pulsed radiofrequency procedure

The PRF was performed in the same way as the diagnostic injection. Standard monitors such as an electrocardiogram, blood pressure, and pulse oximeter were attached, and intravenous access was secured. A 22-gauge 5-cm insulated radiofrequency needle with a 2-mm active tip was introduced akin to the previous diagnostic injection procedure, and the styloid process was contacted under direct fluoroscopic vision. (Figures 4-6). Then the needle was walked off posteriorly from the styloid process. Once bony contact was lost, the contrast was injected to demonstrate local filling and lack of vascular uptake. Sensory stimulation up to 0.5 V at a frequency of 50 Hz reproduced concordant pain at the base of the tongue, pharynx, and ear. Motor stimulation was performed subsequently up to 2.0 V at a frequency of 2 Hz to look for contractions of the neck muscles and diaphragm, which was not elicited. Then 1 mL of lidocaine 2% was injected, and PRF lesioning was performed for three cycles of 90 seconds at a constant temperature of 42°C with a frequency of 2 Hz. After lesioning, 1 mL of 2% lignocaine with dexamethasone 2 mg was injected. The patient remained hemodynamically stable throughout the procedure. There were no adverse events or complications observed after the procedure. He reported a decrease in the pain scores consistently following the procedure. At present, he is completely pain-free without any medication for the last 14 months.

## Discussion

One of the causes of secondary glossopharyngeal neuralgia is Eagle’s syndrome, which is characterized by abnormal elongation of the styloid process. The incidence of elongated styloid process is 4% in the general population, of which only 4% are reported to be symptomatic, with a female-to-male ratio of 3:1. It is usually reported in adults after the third decade of life [4].

Several theories have been postulated as the cause of Eagle’s syndrome, though its exact cause remains unknown. The etiopathogenesis is classically described as compression or straining of the neurovascular structures present in the retrostyloid compartment due to the formation of scar tissue after tonsillectomy surgery. However, Eagle’s syndrome was also seen in a patient who had never been operated before. Embryologically, the styloid process, stylohyoid ligament, and lesser horn of the hyoid bone originate from the Reichert’s cartilage of the second branchial arch. It is theorized that the ossification of remnants of Reichert’s cartilage could be the cause of the elongated styloid process. It is also proposed that the ossification of ligaments due to endocrinological changes after menopause in women is one of the reasons for Eagle's syndrome, perhaps explaining the female preponderance of the disease. The ossified stylohyoid ligament contracts the stylopharyngeus muscle, stretching the cranial nerve IX and triggering the symptoms. [5].

In the original description by Eagle, there were two types of clinical presentation: the stylohyoid and the stylocarotid syndromes. In the former, patients present with continuous pain in the pharyngeal area radiating to the ipsilateral ear due to impingement of the cranial nerve IX, and in the latter, the symptoms are more vascular with pain radiating more to the head and the orbit due to pressure on the internal carotid artery [7]. Our patient certainly belongs to the former category, and the likely etiology might be the degenerative changes in the stylohyoid ligament associated with aging as there was no history of any surgery or trauma.

The diagnosis of Eagle's syndrome requires a high index of suspicion. It is a diagnosis by exclusion and needs to be differentiated from several other conditions, which include otitis media, trigeminal neuralgia, idiopathic glossopharyngeal neuralgia, temporomandibular joint pain, impacted third molar, and neoplasms of the pharynx and tongue. A simple radiograph of the skull (Towne’s view) is helpful in its diagnosis. However, three-dimensional CT scan is the gold standard in diagnosing this condition [8].

Conservative medical management is the first line of management and usually precedes any interventional or surgical therapy. Anticonvulsants such as carbamazepine and gabapentinoids are the preferred initial drugs, which are often supplemented with SSRIs (selective serotonin reuptake inhibitors), opioids, and NSAIDs [9]. Medical management often does not give desired results, and frequently patients have side effects such as dizziness, drowsiness, sedation, and dryness of the mouth [10]. For patients who do not respond to medical management or show intolerable side effects, surgery in the form of styloidectomy is performed either through the extraoral or transpharyngeal route. However, surgical interventions are not straightforward, as the operating site contains many vital structures including major vessels and several cranial nerves (VII, IX, X, XI, and XII). The transpharyngeal or intraoral approach has the disadvantage of poor surgical exposure, lack of control over the major vessels, and possible bacteriological contamination of deep neck spaces. The extraoral route is a safer approach, but it has a longer duration of surgery [11].

A middle path in the form of minimally invasive interventional techniques can play an important role in the management of Eagle's syndrome. Glossopharyngeal nerve block has been employed as both diagnostic and therapeutic measures in the management of glossopharyngeal neuralgia [12]. On confirmation of acceptable pain relief with a diagnostic glossopharyngeal nerve block, percutaneous thermal radiofrequency ablation (RFA) of the nerve is usually the next step to achieve a long-duration effect [13]. PRF treatment is usually preferred over conventional RFA in this scenario as PRF denervation is less destructive and usually not associated with neuritis, motor deficits, or deafferentation pain, unlike the latter one. In PRF denervation, the temperature is kept at 42°C, and studies found that temperature less than 45°C does not cause any irreversible damage to the nerve [14].

There was only one previous case report describing a successful treatment of Eagle's syndrome by PRF of the glossopharyngeal nerve [6]. Moreover, this modality of treatment has been implemented to treat several cases of glossopharyngeal neuralgia of various etiologies [15-16]. It is a relatively safe procedure, but serious complications may occur. There is a possibility of inadvertent injury to the surrounding vessels such as the carotid artery and internal jugular vein leading to the development of hematoma. Besides that, trauma to the adjacent nerves (vagus, hypoglossal, spinal accessory nerve) may cause hemodynamic disturbances, upper airway obstruction, and trapezius weakness [17].

Moreover, conventional RFA and even chemical neurolysis of the glossopharyngeal nerve had been reported to be carried out to manage a resistant case of glossopharyngeal neuralgia [18]. According to the author, these modalities should be performed with great caution in benign pain syndromes, and especially chemical neurolysis should only be considered in painful malignant conditions.

Apart from the glossopharyngeal nerve block and neurolysis, there are descriptions of other novel interventions to manage pain in Eagle’s syndrome. Spinal cord stimulation was reported to be employed in the alleviation of pain in a case of Eagle’s syndrome, who was refractory to both medical and surgical management [19]. In another case, the stellate ganglion block was used in addition to pharmacotherapy to control pain [20]. These novel techniques, particularly the spinal cord stimulation, can be an additional minimally invasive option in selected cases, though further evidence is needed.

In our case, the initial clinical presentations were misleading as the predominant symptom was ear ache. The diagnosis of Eagle’s syndrome was confirmed only after radiological investigations. Presumably, the glossopharyngeal nerve was strained due to the anomalous styloid process as the symptoms were mimicking atypical glossopharyngeal neuralgia. Hence, there was a remarkable reduction in pain due to the glossopharyngeal nerve block and subsequent PRF treatment.

## Conclusions

Eagle’s syndrome is a rare cause of craniofacial pain characterized by an elongated styloid process. The clinical presentation often mimics features of glossopharyngeal neuralgia. Management option includes pharmacological, surgical, and minimally invasive interventions. PRF treatment of the glossopharyngeal nerve can be an effective, safe, and minimally invasive management option for providing pain relief in selected symptomatic cases.
